# Influence of Umbelliferone on the Anticonvulsant and Neuroprotective Activity of Selected Antiepileptic Drugs: An In Vivo and In Vitro Study

**DOI:** 10.3390/ijms23073492

**Published:** 2022-03-23

**Authors:** Mirosław Zagaja, Anna Zagaja, Joanna Szala-Rycaj, Aleksandra Szewczyk, Marta Kinga Lemieszek, Grzegorz Raszewski, Marta Andres-Mach

**Affiliations:** 1 Isobolographic Analysis Laboratory, Institute of Rural Health, Jaczewskiego 2, 20-090 Lublin, Poland; szala-rycaj.joanna@imw.lublin.pl (J.S.-R.); szewczyk.aleksandra@imw.lublin.pl (A.S.); andres.marta@imw.lublin.pl (M.A.-M.); 2 Chair and Department of Humanities and Social Medicine, Medical University of Lublin, Chodźki 7, 20-090 Lublin, Poland; 3 Department of Medical Biology, Institute of Rural Health, Jaczewskiego 2, 20-090 Lublin, Poland; lemieszek.marta@imw.lublin.pl; 4 Department of Toxicology and Food Protection, Institute of Rural Health, Jaczewskiego 2, 20-950 Lublin, Poland; raszewskigj@gmail.com

**Keywords:** umbelliferone, epilepsy, psychomotor seizures, drug interactions, neuroprotection, trophic stress, excitotoxicity

## Abstract

Umbelliferone (7-hydroxycoumarin; UMB) is a coumarin with many biological properties, including antiepileptic activity. This study evaluated the effect of UMB on the ability of classical and novel antiepileptic drugs (e.g., lacosamide (LCM), levetiracetam (LEV), phenobarbital (PB) and valproate (VPA)) to prevent seizures evoked by the 6-Hz corneal-stimulation-induced seizure model. The study also evaluated the influence of this coumarin on the neuroprotective properties of these drugs in two in vitro models of neurodegeneration, including trophic stress and excitotoxicity. The results indicate that UMB (100 mg/kg, i.p.) significantly enhanced the anticonvulsant action of PB (*p* < 0.01) and VPA (*p* < 0.05), but not that of LCM orLEV, in the 6-Hz test. Whether alone or in combination with other anticonvulsant drugs (at their ED_50_ values from the 6-Hz test), UMB (100 mg/kg) did not affect motor coordination; skeletal muscular strength and long-term memory, as determined in the chimney; grip strength; or passive avoidance tests, respectively. Pharmacokinetic characterization revealed that UMB had no impact on total brain concentrations of PB or VPA in mice. The in vitro study indicated that UMB has neuroprotective properties. Administration of UMB (1 µg/mL), together with antiepileptic drugs, mitigated their negative impact on neuronal viability. Under trophic stress (serum deprivation) conditions, UMB enhanced the neurotrophic abilities of all the drugs used. Moreover, this coumarin statistically enhanced the neuroprotective effects of PB (*p* < 0.05) and VPA (*p* < 0.001) in the excitotoxicity model of neurodegeneration. The obtained results clearly indicate a positive effect of UMB on the anticonvulsant and neuroprotective properties of the selected drugs.

## 1. Introduction

Epilepsy, a chronic disease that results in neurobiological, cognitive, psychological and social dysfunctions, significantly reduces patients’ quality of life [1]. Despite the increasing number and variety of anti-seizure medications (ASMs), approximately 30–40% of epilepsy patients suffer from side effects from the already-administered drugs, including drowsiness, fatigue, dizziness, unsteadiness, blurred or double vision, concentration deficits, memory problems, irritability, and depression [2]. Furthermore, both seizures and the antiepileptic drugs themselves can cause neurodegeneration, e.g., damage to nerve cells [3]. Therefore, it is extremely important that the used drugs also exhibit neuroprotective properties, a trait that is not characteristic to all ASMs [4,5,6]. Because of this, there is an urgent need to find active compounds (synthetic or natural) that exhibit better antiseizure and neuroprotective properties, as well as lower toxicity. Natural compounds that possess such properties—i.e., that have antiepileptic properties, and also increase the pharmacological activity of the already known ASMs—include coumarins [7,8,9,10,11,12,13,14,15].

Coumarins are a commonly existing group of secondary plant metabolites found in 30 different families of higher plants, but mainly in Rutaceae and Umbelliferae. They are found in all plant parts (fruits, seeds, roots, stems and leaves) and fulfill numerous functions, including growth regulation and bactericidal and fungicidal activity. There are over 1300 identified coumarins that exhibit many biological and pharmacological activities [16,17,18]. A very interesting compound belonging to this group is umbelliferone (7-hydroxycoumarin, UMB), commonly found in the leaf, roots and fruits of the Umbelliferae family; chemically, it is a benzopyrone in nature [19].

UMB has been reported to exhibit anti-inflammatory and antioxidant activity in a chronic animal model of alcohol-fed rats and streptozotocin-induced diabetes [20,21]. Furthermore, anti-cancer and anti-apoptotic activity of UMB has also been documented in various models of carcinogenesis, i.e., colon, hepatic and renal cancer [22,23,24]. This coumarin is also known for its antiarthritic [25], hepatoprotective [26], anti-asthmatic [27], and anti-allergic activity (preclinical studies) [28]. UMB also has antinociceptive properties in different models of pain, such as acetic-acid-induced writhing, the formalin test, and complete Freund’s adjuvant-induced hyperalgesia [29,30].

It is worth noting that UMB has protective potential in neurodegenerative diseases. UMB reportedly exhibits neuroprotective properties in a chronic, unpredictable, mild-stress-induced rat model of depression [31], a rat model of focal cerebral ischemia–reperfusion [32] and a mouse model of Parkinson’s disease [33]. It also possesses procognitive properties in STZ-induced cognitive dysfunction in rats. This effect may be attributed, at least partially, to inhibiting acetylcholinesterase and attenuating oxidative stress, neuroinflammation, and neuronal loss [34].

In vitro studies reveal that UMB exhibits both antioxidant and neuroprotective properties [35,36]. Furthermore, it can be administered safely and effectively because it is non-toxic in low doses and easily crosses the blood–brain barrier [37]. Due to its simple structure, it is used as a parent compound in the synthesis of various coumarins and heterocyclic compounds [38].

Furthermore, our previous research using UMB and selected ASMs demonstrated its anticonvulsant properties in the maximum-electric-shock test (MES) in mice—an animal model of tonic–clonic seizures in humans [12]. In this research, UMB administered at a dose of 150 mg/kg significantly increased the seizure threshold in the maximal electroshock-seizure threshold test in mice (approximately 37%). Moreover, it significantly increased the anticonvulsant effects of phenobarbital (PB) and valproic acid (VPA), two classic ASMs. In addition, no effect of UMB on the total concentration of these drugs in the brains of the test animals was demonstrated, which indicates the pharmacodynamic profile of the interaction between this coumarin and the above-mentioned drugs [12].

Taking into account the obtained data resulting from the use of UMB and selected ASMs, it is extremely important to continue the research and extend it to another model of experimental epilepsy, which is the 6-Hz corneal stimulation model; this is considered to be an experimental model of psychomotor (limbic) seizures in humans. More specifically, various stereotypical and behavioral manifestations of seizure activity in animals subjected to the electrical stimulation, with the current at frequency of 6 Hz, are similar to those observed in patients with partial (limbic) seizures [39,40].

Therefore, the use of this animal model of epilepsy and the assessment of the nature of the interaction between UMB and the selected antiepileptic drugs (lacosamide (LCM), levetiracetam (LEV), phenobarbital (PB) and valproate (VPA)) seems justified. The drug selection was based on their various different molecular mechanisms of action, and their specific action in the 6-Hz corneal stimulation model. Additionally, to determine the acute adverse-effect profiles for the combinations of UMB with LCM, LEV, PB and VPA, three behavioral tests (chimney, passive avoidance, and grip-strength) were used. To confirm or exclude any pharmacokinetic background for the observed interactions between UMB and the studied antiepileptic drugs, total brain concentrations of antiepileptic drugs were measured using the HPLC techniques. Furthermore, taking into consideration the protective properties of UMB, an analysis of the effect of this coumarin on the neuroprotective properties of selected ASMs in in vitro models was performed. The direct effect of the tested ASMs and their combination with UMB on the viability of nerve cells was determined in two models of neurodegeneration, including trophic stress, caused by the removal of B27 Supplement from the culture medium, and excitotoxicity, caused by glutamate. The conducted research certainly contributes to the broadening of knowledge on the possibility of enhancing the anticonvulsant/neuroprotective effect of ASMs by combining it with a natural substance such as UMB. Additionally, the obtained results may be important in the context of drug safety.

## 2. Results

### 2.1. Influence of UMB on the Anticonvulsant Activity of Various ASMs in the 6-Hz Corneal-Stimulation-Induced Seizure Model in Mice

**Figure 1:**
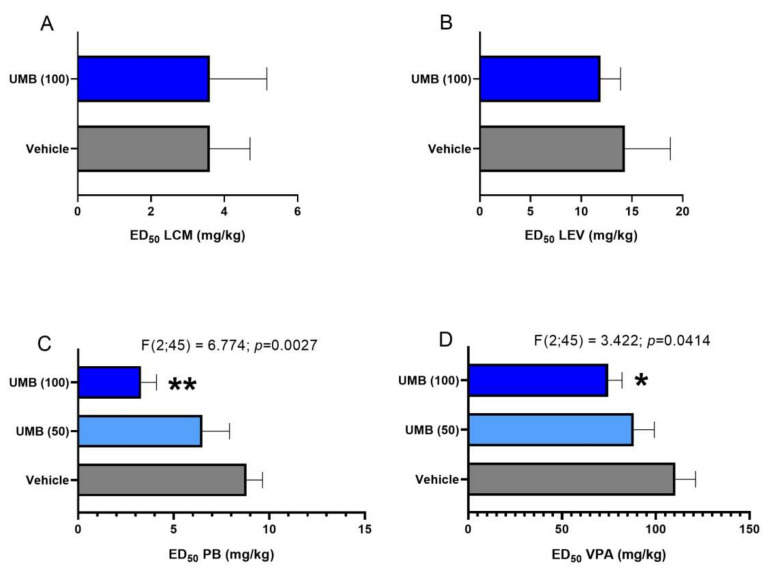
(**A**–**D**) Effects of UMB on the anticonvulsant potency of CBZ, LCM, LTG and VPA in the 6 Hzcorneal-stimulation-induced seizure model in mice. Columns represent median effective doses (ED_50_ in mg/kg ± SEM) of antiepileptic drugs (LCM (**A**), LEV (**B**), PB (**C**) and VPA (**D**)) that protected half of the tested mice from psychomotor seizures. The log-probit method was used for calculating the ED_50_ values (each experimental group consisted of 8 mice). ** *p* < 0.01, * *p* < 0.05 vs. control (PB, VPA + vehicle-treated) animals (one-way ANOVA and post-hoc Tukey–Kramer test).

When administered alone, LCM, LEV, PB and VPA protect the experimental animals, in a dose-dependent manner, from the 6-Hz corneal stimulation seizures. Their ED_50_ values are presented in Figure 1A–D. UMB (100 mg/kg) co-administered with PB significantly enhanced the anticonvulsant effect of the latter against the 6-Hz corneal stimulation model [F(2;45) = 6.774; *p* = 0.0027], by reducing its ED_50_ value from 8.8 mg/kg to 3.3 mg/kg (by 62.5%; *p* < 0.01) (Figure 1C). UMB at a lower dose of 50 mg/kg did not significantly potentiate the anti-seizure activity in the 6-Hz corneal stimulation model (Figure 1C). 

With regard to VPA, UMB at a dose of 100 mg/kg markedly potentiated the anticonvulsant effects of this drug in the 6 Hz-induced psychomotor seizure test [F(2;45) = 3.422; *p* = 0.0414], by decreasing its ED_50_ value from 110.5 mg/kg to 74.6 mg/kg (by 22.5%; *p* < 0.05) (Figure 1D). However, UMB at the lower dose of 50 mg/kg had no significant influenceon the antiepileptic effect of VPA in this experimental seizure model (Figure 1D).

UMB at doses of 100 mg/kg did not significantly affect the anticonvulsant effect of LCM and LEV in the 6 Hz-induced psychomotor seizure test (Figure 1A,B).

### 2.2. Effects of UMB in Combination with the Studied ASMs on Motor Performance, Muscular Strength and Long-Term Memory in Mice

UMB (ata dose of 100 mg/kg) administered in combination with LCM (3.6 mg/kg), LEV (11.9 mg/kg), PB (3.3 mg/kg) and VPA (74.6 mg/kg) significantly changedneither the skeletal muscular strength nor the motor coordination in the animals subjected to the grip-strength and chimney tests, respectively (Table 1). Moreover, UMB in combination with these drugs did not markedly disturb the learning and remembering processes in experimental animals subjected to the passive avoidance task (Table 1).

Withregard to ASMs administered alone at doses corresponding to their ED_50_ values from the 6Hz test, the antiepileptic drugs had no significant impact on motor performance, skeletal muscular strength, orlong-term memory in mice (Table 1).

### 2.3. Effect of UMB on Total Brain Antiepileptic Drug Concentrations

**Figure 2:**
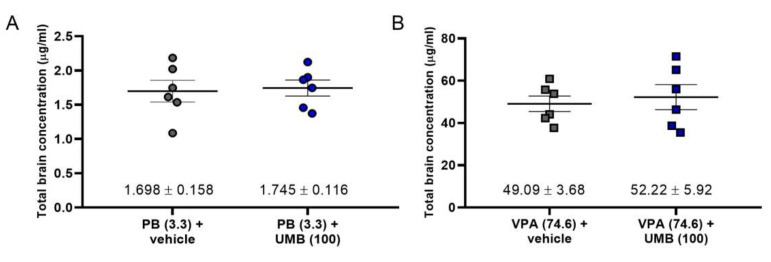
(**A**,**B**) Influence of UMB on total brain concentrations of classical ASMs in mice. Scatter plots represent total brain concentrations of PB and VPA in µg/mL (as means ± SEM, as the error bars; n = 6 mice/group). No statistical significance between the means was observed (unpaired Student’s *t*-test).

The total brain concentration of PB (3.3 mg/kg) administered alone was 1.698 ± 0.158 μg/mL and did not significantly differ from that determined for the combination of PB (3.3 mg/kg) with UMB (100 mg/kg), which was 1.745 ± 0.116 μg/mL. Similarly, the total brain concentration of VPA (74.6 mg/kg) administered separately was 49.09 ± 3.68 μg/mL and did not significantly differ from that determined for the combination of VPA (74.6 mg/kg) with UMB (100 mg/kg), which was 52.22 ± 5.92 μg/mL (Figure 2). 

Since UMB at 100 mg/kg did not significantly affect the anticonvulsant potential of LCM and LEV in the 6 Hz test, the total brain concentrations of this drug were not measured.

### 2.4. The UMB Influence on Neuron Viability

**Figure 3:**
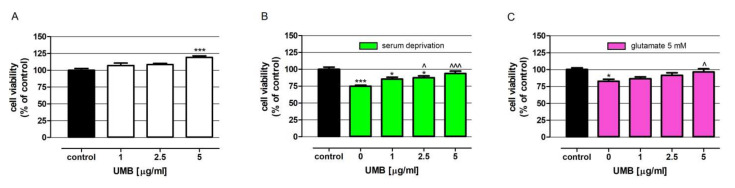
Influence of UMB on the viability of neurons under standard or degenerative conditions: trophic stress (serum deprivation) and glutamate excitotoxicity (5 mM glutamate). Neurons derived from human neuroblastoma cell line SH-SY5Y were exposed for 48 h to UMB at concentrations 1, 2.5 and 5 μg/mL prepared in culture medium alone (control) (**A**), or serum-deprived cell culture medium (**B**) or with 5 mM glutamate (**C**). Cell viability was measured photometrically using an MTT assay. Results are presented as the mean of at least 4 measurements. Statistically significant differences compared to the control at * *p* < 0.05, *** *p* < 0.001; statistically significant differences compared to the degenerative conditions at ^ *p* < 0.05, ^^^ *p* < 0.001. One-way ANOVA test and Tukey’s post-hoc test.

UMB at the highest tested concentration (5 µg/mL) increased neuron metabolic activity by 19.2% (Figure 3A). Studies conducted under trophic stress conditions revealed that UMB significantly weakened the negative impact of serum deprivation on neuronal cell viability. UMB at concentrations of 2.5 and 5 µg/mL increased neuron metabolic activity from 74.9% to 87.6% and 93.8%, respectively (Figure 3B). As presented in Figure 3C, UMB at the highest tested concentration almost completely neutralized the toxic effect of 5 mM glutamate, which decreased neuron metabolic activity by 17.5%.

### 2.5. Influence of Various ASMs and Their Combination with UMB on the Viability of Neurons

**Figure 4:**
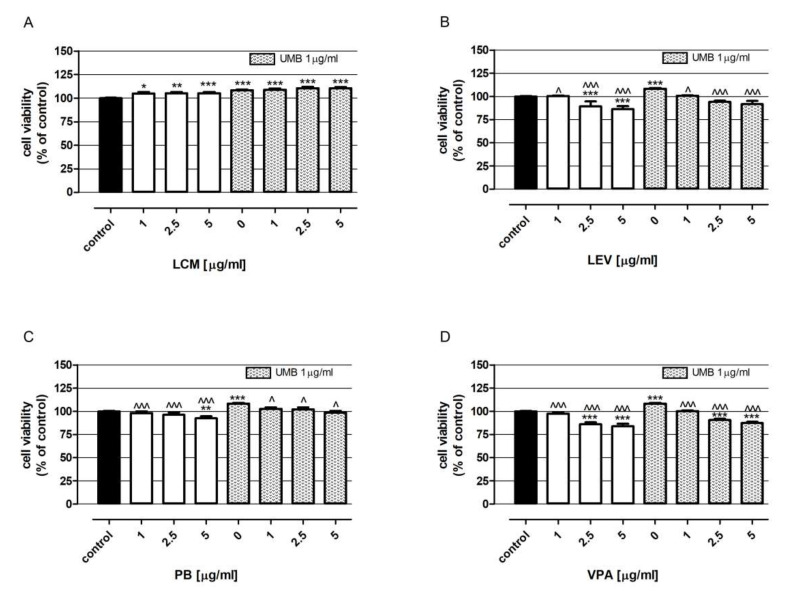
UMB impact on the biological activity of LCM (**A**), LEV (**B**), PB (**C**) and VPA (**D**) in neurons derived from human neuroblastoma cell line SH-SY5Y. Cells were exposed for 48 h to investigated drugs at concentrations 1, 2.5, and 5 μg/mL prepared with or without 1 μg/mL UMB. The control consisted of cells exposed to culture medium alone. Cell viability was measured photometrically using an MTT assay. Results are presented as the mean of at least 4 measurements. Statistically significant differences compared to the control at * *p* < 0.05, ** *p* < 0.01, *** *p* < 0.001;statistically significant differences compared to the UMB at ^ *p* < 0.05, ^^^ *p* < 0.001. One-way ANOVA test and Tukey’s post-hoc test.

Out of all of the tested drugs, only LCM increased the viability of neurons, and the strongest stimulation (5.4%) of cells’ metabolic activity was observed after incubation with 5 μg/mL LCM (Figure 4A). On the contrary, other tested drugs decreased neurons’ viability in a dose-dependent manner. Cells’ exposure to LEV, PB and VPA at the highest tested concentrations (5 μg/mL) lowered neurons’ viability by 13.8%, 7.4% and 16.0%, respectively (Figure 4B–D). UMB’s influence on neurons’ viability was similar to the effect observed after cells’ treatment with LCM. In contrast, UMB used alone revealed a significantly stronger beneficial effect than the other tested compounds.

Adding UMB at concentrations of 1 μg/mL mitigated the negative effect of LEV, PB and VPA on the viability of nerve cells (Figure 4 B–D). 

### 2.6. Influence of UMB on the Neuroprotective Effect of Various ASMs under Trophic Stress Conditions

**Figure 5:**
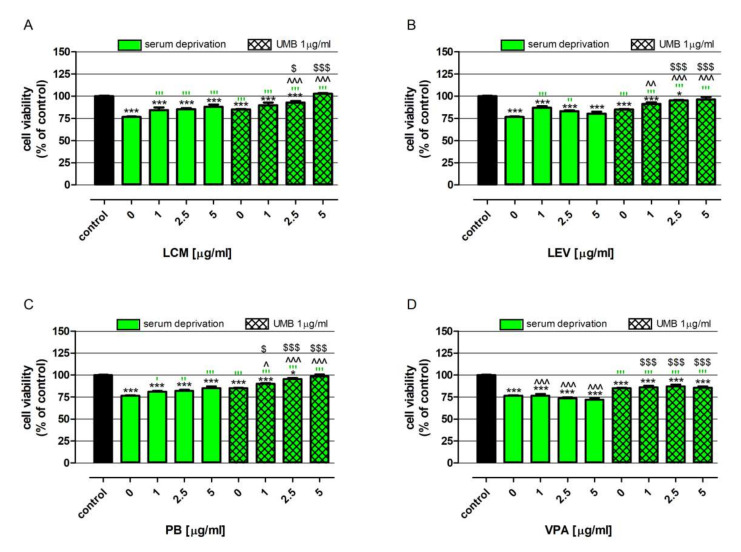
UMB impact on the biological activity of LCM (**A**), LEV (**B**), PB (**C**) and VPA (**D**) in neurons under trophic stress (serum deprivation) conditions. Neurons derived from human neuroblastoma cell line SH-SY5Y were exposed for 48 h to investigated drugs at concentrations 1, 2.5, and 5 μg/mL prepared in serum-deprived medium with or without 1 μg/mL UMB. The control consisted of cells exposed to culture medium alone. Cell viability was measured photometrically using as MTT assay. Results are presented as the mean of at least 4 measurements. Statistically significant differences compared to the control at * *p* < 0.05, *** *p* < 0.001; statistically significant differences compared to the UMB at ^ *p* < 0.05, ^^ *p* < 0.01, ^^^ *p* < 0.001; statistically significant differences compared to the serum-deprivation medium at ′ *p* < 0.05, ″ *p* < 0.01, ‴ *p* < 0.001; statistically significant differences between neurons treated with drug vs. neurons exposed to both UMB and drug at the corresponding concentrations at $ *p* < 0.05, $$$ *p* < 0.001. One-way ANOVA test and Tukey’s post-hoc test.

As presented in Figure 5, serum deprivation decreased neurons’ mitochondrial activityby 23.4% compared to the control (neurons cultured in medium with B27 supplement). Among the investigated drugs, only VPA did not impact neurons’ viability under trophic stress conditions (Figure 5D). On the contrary, LCM and PB in the whole range of tested concentrations (Figure 5A,C), while LEV, at concentrations 1 and 2.5 µg/mL, effectively increased cells’ viability, which was lowered by serum deprivation (Figure 5B). It needs to be highlighted that the beneficial effect of LCM and PB intensified with the increase in the concentration of the tested substances; consequently, neurons’ viability, in response to the mentioned drugs at a concentration of 5µg/mL, rose from 76.6% to 88.0% and 85.1%, respectively. In the case of LEV, the strongest neuroprotective effect was observed in cells treated with 1 µg/mL, wherein neurons’ viability increased from 76.6% to 87.0% (Figure 5B). 

Furthermore, it also needs to be stressed that the drugs’ impact on neurons’ viability improved in the presence of UMB. Favorable changes were observed in cells exposed to UMB together with PB and VPA in the whole range of tested concentrations, as well as for those exposed to LCM and LEV at concentrations 2.5 and 5 µg/mL (Figure 5). The comparison data obtained from the drugs used alone, at a concentration of 5 µg/mL, with drugs at the corresponding concentration, administered together with UMB, revealed an increase in neuron viability of 14.7% (LCM + UMB vs. LCM), 16.2% (LEV + UMB vs. LEV), 13.4% (PB + UMB vs. PB), and 13.5% (VPA + UMB vs. VPA). 

### 2.7. Influence of UMB on the Neuroprotective Effect of Various ASMs in Excitotoxicity Model of Neurodegeneration

**Figure 6:**
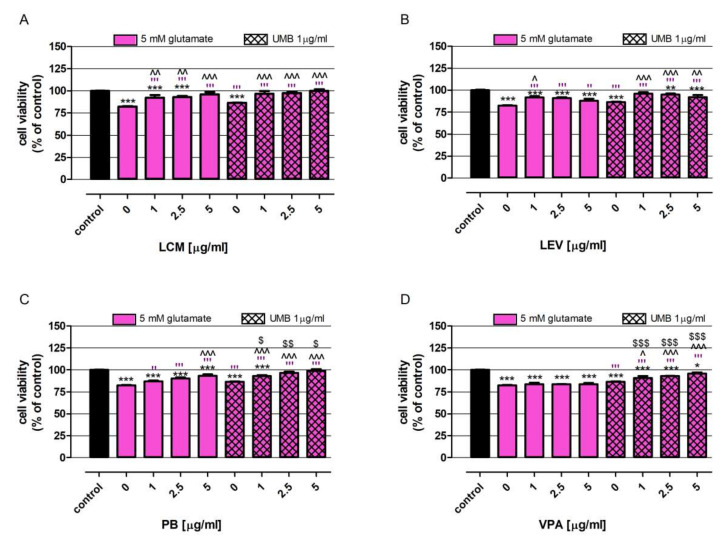
UMB impact on the biological activity of LCM (**A**), LEV (**B**), PB (**C**) and VPA (**D**) in neurons under glutamate excitotoxicity conditions. Neurons derived from human neuroblastoma cell line SH-SY5Y were exposed for 48 h to investigated drugs at concentrations 1, 2.5, and 5 μg/mL in glutamate medium (5 mM glutamate), with or without 1 μg/mL UMB. The control consisted of cells exposed to culture medium alone. Cell viability was measured photometrically using an MTT assay. Results are presented as the mean of at least 4 measurements. Statistically significant differences compared to the control at * *p* < 0.05, ** *p* < 0.01, *** *p* < 0.001; statistically significant differences compared to the UMB at ^ *p* < 0.05, ^^ *p* < 0.01, ^^^ *p* < 0.001; statistically significant differences compared to the glutamate medium at ″ *p* < 0.01, ‴ *p* < 0.001; statistically significant differences between neurons treated with drug vs. neurons exposed to both UMB and drug at the corresponding concentrations at $ *p* < 0.05, $$ *p* < 0.01, $$$ *p* < 0.001. One-way ANOVA test and Tukey’s post-hoc test.

As presented in Figure 6, glutamate at a concentration of 5 mM decreased neurons’ viability by 17.4%. VPA was not able to protect neurons from the excitotoxic effect of glutamate (Figure 6D). However, LCM, PB and VPA, in the whole range of tested concentrations, significantly increased the viability neurons lowered by glutamate (Figure 6A–C). The beneficial effect of LCM and PB intensified with the increase in their concentrations; the mentioned drugs at a concentration of 5 µg/mL elevated neurons’ viability from 82.4% to 96.0% and 93.2%, respectively (Figure 6A,C). In the case of LEV, its neuroprotective effect was inversely correlated with its concentration; consequently, the best neuron response was observed after cell treatment with a dose of 1 µg/mL, wherein neurons viability was 91.6% (Figure 6B). 

UMB at a concentration of 1 µg/mL also revealed a beneficial impact on neurons affected by glutamate treatment; the tested coumarin increased cell viability by 4.1%. The discovered neuroprotective abilities of UMB in an excitotoxic model of neurodegeneration were weaker than the effect of LCM (1, 2.5, 5 µg/mL), LEV (1 µg/mL) and PB (5 µg/mL) (Figure 6A–C). 

Furthermore, the neuroprotective effects of the combinations PB + UMB and VPA + UMB were significantly stronger than the changes induced by these drugs used separately. The comparison data obtained from the mentioned drugs, used alone at a concentration of 5 µg/mL with drugs at corresponding concentrations and administered together with UMB, revealed an increase in neuron viability of 5.5% (PB + UMB vs. PB) (Figure 6C) and 12.1% (VPA + UMB vs. VPA) (Figure 6D). 

## 3. Discussion

The aim of this study was to assess the influence of UMB on the protective properties of LEV, LCM, PB and VPA in the 6-Hz corneal-stimulation-induced seizure model in mice. The combination of UMB and selected ASMs allowed for the determination of its impact on increasing the anticonvulsant effectiveness of the studied drugs. For combinations in which an increase in the protective effect was found, the type of interaction, as well as the determination of whether the interaction was pharmacodynamic or pharmacokinetic, was investigated. Additionally, an analysis of the influence of this coumarin on the neuroprotective properties of the selected ASMs in two in vitro models of neurodegeneration, including trophic stress and excitotoxicity, was conducted.

UMB (100 mg/kg) increased the anticonvulsant effect of PB and VPA, as shown in the results by a statistically significant reduction in their ED_50_ values. A slight decrease in ED_50_ was also observed for the combination of UMB + LCM and UMB + LEV, but without a significant statistical effect. Similar results for PB and VPA were presented by Zagaja et al. [12] using the mouse MES model for the combination of UMB with selected classic ASMs. XANT administered at a dose of 150 mg/kg in combination with VPA significantly increased the anticonvulsant effect of VPA by reducing the effective dose from 281.4 mg/kg to 215.5 mg/kg. In addition, UMB administered intraperitoneally at 150 mg/kg, significantly enhanced PB anticonvulsant activity, decreasing the ED_50_ of this drug from35.39 to21.78 mg/kg. However, UMB (150 mg/kg, i.p.) did not affect the protective effects of carbamazepine (CBZ) and phenytoin (PHT) on seizures induced by MES in mice [12]. 

Moreover, our studies of epilepsy in animal models show anticonvulsant properties in the following coumarins: imperatorin [41], osthol [42], XANT [11], and UMB [12]. In addition, we showed a beneficial interaction between the aforementioned coumarins and classical antiepileptic drugs in the maximum-electric-shock test (MES) in mice. Imperatorin increased the paroxysmal activity of CBZ, PB, PHT [7] and lamotrigine [8]. In turn, XANT statistically increased the anticonvulsant properties of as many as six antiepileptic drugs, both classic (VPA and CBZ) and new generation: LCM, oxcarbazepine (OXC), pregabalin (PGB) and topiramate (TPM) [13,14]. Our latest research on coumarins studied the effects of XANT on selected antiepileptic drugs in the same model, i.e., the 6-Hz corneal stimulation model in mice. XANT (50 mg/kg, administered i.p.) significantly potentiated the anticonvulsant activity of LEV and VPA. Furthermore, XANT had no impact on LEV total brain concentrations, indicating the pharmacodynamic nature of interactions between these antiepileptic drugs in this model [15].

The intensification of PB and VPA’s anticonvulsant activity under the influence of UMB, observed in the conducted studies, most likely results from the synergistic effect of the tested substances on molecular pathophysiological mechanisms. To explain the synergy of the mixture of PB and VPA with UMB, one should consider the molecular mechanisms of action of the used ASMs. The main mechanism of activity of both of these drugs is based on GABA-ergic inhibition enhancement.

Regarding PB, the drug shows an antiepileptic effect based on binding to the GABAA receptor, enhancing the inhibitory effect on the nervous system and prolonging the opening time of chloride channels [43,44]. Barbiturates increase both postsynaptic and presynaptic GABA-ergic inhibition, which is not associated with an increase in the frequency of chloride channels opening, but occurs through a marked extension of their opening time [45]. PB also inhibits calcium and sodium channels, and these actions may contribute to the therapeutic activity of this drug [46]. The mechanism of the antiepileptic action of VPA is very complex; however, the activity of this drug most likely results from the GABA mechanism. It includes changes in the concentration of the GABA neurotransmitter and an increase in its concentration in the brain, possibly by inhibiting GABA degradative enzymes such as GABA transaminase and succinate-semialdehyde dehydrogenase, and by inhibiting the re-uptake of GABA by neuronal cells. It affects the potassium and sodium channels of the neuronal membrane; reducing its excitability and regulating voltage changes; delaying the reactivation of sodium channels; and modifying calcium transmission. VPA, in addition to enhancing inhibitory GABA-ergic transmission, raises the excitability threshold by modulating intracellular signaling pathways [47,48].

According to the literature, coumarin’s antiepileptic activity is the result of the GABA-transaminase activity inhibition and being a partial agonist of the GABA-benzodiazepine. It is known that imperatorin inactivates the GABA degradative enzyme, GABA transaminase, in both a time- and concentration-dependent manner; consequently, it increases the GABA content in the synaptic cells of neurons and elevates the inhibitory neuro-transmitter GABA level in the brain [49,50]. According to Singhuber et al. [51], oxyprenylated coumarin derivatives, such as osthole, oxypeucedanin, and imperatorin, represent a new group of GABA receptor modulators. It has been suggested that prenyl residues are essential for positive modulatory activity of these substances [51]. However, for UMB, such activity has not been confirmed, which is probably due to its structure. Thus, bearing in mind the diverse molecular mechanisms of action of the studied ASMs and UMB, joining them together can evoke synergistic (favorable) interaction in protecting the experimental animals from the mouse 6-Hz corneal stimulation model.

The results obtained from the assessment of the effect of UMB on total PB or VPA concentrations in brain tissue did not show a statistically significant increase in PB or VPA in combination with UMB; this indicates the pharmacodynamic nature of the interaction between the tested substances. In previous studies, UMB exhibited similar activity (in the MES test) [12]. However, XANT (50 mg/kg, i.p.) significantly increased total brain concentrations of VPA, indicating the pharmacokinetic nature of the interactions between drugs in the 6 Hz mouse psychomotor seizure model, which further indicates the pharmacokinetic nature of the interaction between test substances. Similar relationships were observed in studies conducted by Zagaja et al. [13], wherein a pharmacokinetic interaction of VPA and XANT was demonstrated in the MES test. Moreover, in the same study, XANT also increased total CB concentration in the brains of tested animals, without a significant impact on PB and PHT levels. In turn, imperatorin administered at a dose of 30 mg/kg increased the carbamazepine concentration in mouse brains by 85% [7]. 

Assessment of adverse reaction profile in selected behavioral tests for both LEV, LCM, VPA and PB administered separately and in combination with UMB (100 mg/kg), at doses corresponding to their ED_50_ values, did not indicated any adverse effects of the tested combinations. None of the combinations detected significantly impaired motor coordination in the chimney test, skeletal muscle strength in the grip-strength test, or long-term memory in the passive avoidance test. Furthermore, XANT tested alone, at doses of 50 and 100 mg/kg, showed no adverse effects in tested animals in behavioral tests [13,14]. This coumarin, administered at a dose of 50 mg/kg in combination with LEV, LCM, VPA and PB, also showed no adverse effects. However, at a higher dose, XANT (100 mg/kg), in combination with LCM, LTG and PGB, significantly impaired skeletal muscle strength, as seen in the grip-strength test. In the chimney test, PGB in combination with XANT (100 mg/kg) also statistically significantly impaired motor coordination [14]. Of the combinations tested of other coumarins with ASMs, imperatorin with four classical antiepileptic drugs (CBZ PB, PHT, VPA), as well as osthole (150 mg/kg) with CBZ and PB, exhibited no adverse effects in behavioral tests [7,9]. The above studies show that UMB, as well as other coumarins, are well tolerated in most of the combinations with ASMs, without showing acute side effects. 

The neuroprotective properties of the tested substances were evaluated in two in vitro models of neurodegeneration, including trophic stress and excitotoxicity. Studies were conducted in neurons derived from human neuroblastoma cell line SH-SY5Y. These cells were derived from human immature neoplastic neural crest cells that reveal properties of stem cells; because of this, SH-SY5Y cells, using a variety of agents, can be differentiated into neurons. It needs to be highlighted that the types of differentiating agents induce different neuronal phenotypes [52]. Used in the presented studies, retinoic acid induces differentiation toward a cholinergic phenotype and increases the susceptibility of the cells to neurotoxins, which makes this model an excellent tool for assessing the neuroprotective properties of substances. Many studies report that neurons derived from SH-SY5Y cells in response to retinoic acid are a stable population (nonproliferating cells) with extensive neurite outgrowth, as well as morphological and biochemical similarity to living neurons in the human mature brain [53,54,55].

The obtained results indicate the beneficial impact of UMB on neuron viability, as well as neuroprotective properties, especially neurotrophic and anti-excitotoxicity. Studies conducted by Demirkaya et al. [36] also revealed that UMB at concentrations of 25–250 μM showed a neuroprotective effect in primary cortical neurons against glutamate excitotoxicity. Furthermore, total antioxidant status analysis results showed a 50–250 μM UMB increase in the level of antioxidants in cells, which can help protect neurons against glutamate-induced excitotoxicity. It has been indicated that glutamate mediates excitotoxicity in primary cultured neurons by stimulating the N-methyl-d-aspartate receptor which, in turn, leads to increased calcium permeability and the formation of reactive oxygen species (ROS), as well asthe release of lysosomal enzymes [56,57]. Subramaniam and Ellis [33] showed that treatment of animals with UMB decreases caspase 3 activation in dopaminergic neurons, demonstrating that the coumarin prevents apoptotic cell death induced by neurotoxin 1-methyl-4-phenyl-1,2,3,6-tetrahydropyridine (MPTP) in a mouse model of Parkinson’s disease. UMBs’ ability to cross the blood–brain barrier [58], and theirability to scavenge reactive oxygen species (ROSs) directly [59] may be one mechanism by which they contribute to neuroprotection. UMB at physiological pH is among the best hydroperoxyl radical (HOO•)scavengers in water, with activity comparable to that of caffeic acid, but higher than that of ascorbic acid [35]. Among the ROSs, the peroxyl radicals (ROO•) are considered to be a highly toxic species due to their ability to initiate a whole cascade of radical reactions from other organic structures [60]. Moreover, literature data shows that UMB also exerts antioxidative effects, observed as a decrease in myeloperoxidase (MPO) and malondialdehyde (MDA), and increased superoxide dismutase (SOD) activity in the lung tissue of LPS-treated mice. Furthermore, this coumarin also decreases the production of inflammatory cytokines, including monocyte chemotactic protein-1 (MCP-1), IL-6, TNF-α, and IL-1 [61,62].

In our study, it must be noted that LEV, PB and VPA at the highest tested concentrations (5 μg/mL) lowered neuron viability. In turn, LCM, LEV and PB effectively increased cells’ viability, which was lowered by serum deprivation. Moreover, these drugs in the whole range of tested concentrations significantly increased neuron viability, which was lowered by glutamate. Among the investigated drugs, only VPA showed no neuroprotective properties in either of the in vitro neurodegeneration models.

Additionally, in the case of the studied drugs, literature on the subject confirms their neuroprotective properties. Neuroprotective properties have been demonstrated for LCM in a gerbil cerebral ischemia model [63]. The results of the conducted study showed that pre- and postoperative treatment of gerbils with LCM (25 mg/kg) had a protective effect on CA1 neuronal pyramidal cells in the hippocampus of tested animals. Interestingly, also in the in vitro model of ischemia, LCM protects striatal and hippocampal neurons from degeneration [64]. Moreover Nirwan et al. [65] showed that LCM (20 and 40 mg/kg) protected against PILO-induced status epilepticus in C57BL/6 mice, while preventing neurodegeneration and spatial memory impairment. A number of in vitro and in vivo studies indicate the neuroprotective properties of LEV. Sendrowski et al. [66] showed that pre-conditioning of hippocampal cultures with high concentrations of LEV (100 mM and 300 mM) protects neurons against hypoxia-induced death. Such an effect was not observed in the case of lower LEV doses. Oliveira et al. [67] showed that LEV (200 mg/kg) protects neurons and counteracts oxidative stress in mouse hippocampus after PILO-induced seizures (400 mg/kg). Similarly, other studies using LEV in a lithium-pilocarpine epilepsy model in rats have shown that 5-day administration of this drug (150 mg/kg) reduces neuronal damage in test animals [68].

PB, in turn, is a drug that possesses both neuroprotective and neurodegenerative properties. In the study carried out by Vizuete et al. [69], PB exhibited protective effects against 1-methyl-4-phenylpyridinium ion (MPP+)-induced toxicity in rats. PB turned out to also block neuronal cell death after exposure to kainic acid [70]. Rekling [4] proved that PB protects cells in hippocampal slice cultures from death induced by oxygen/glucose deprivation (OGD), which is an in vitro model that simulates cerebral ischemia. Quite surprisingly, the study by Fishman et al. [71] demonstrates a 20–30% reduction in cerebellar Purkinje- and granule-cell count after exposure to PB early in life. Contrary to the results of our studies, Bebitoğlu et al. [72] researched the effects of VPA against glutamate excitotoxicity in the SH-SY5Y human neuroblastoma cell line, and revealed that 1 mM VPA was effective in increasing the viability of cells exposed to glutamate for 24 h; however, at higher doses, this effect was no longer observed. Oxidative damage was observed in SH-SY5Y cells treated with glutamate and was reduced by pre-treatment with VPA. The authors suggested that VPA may exert an anti-oxidant effect against glutamate-induced excitotoxicity. In turn, Rekling [4] showed that VPA does not possess neuroprotective propertiesin an in vitro model of cerebral ischemia. In turn, in in vivo studies, VPA protected neurons from intracerebral hemorrhage [73] and ischemic stroke [74].

Numerous data, including ours, show that the tested ASMs have neuroprotective properties; however LEV, PB and VPA decreased the viability of nerve cells. Our results show that UMB administration—probably due to its neuroprotective properties—together with these drugs mitigates the negative effects of the mentioned drugs on neurons’ viability. In addition, UMB enhances the neurotrophic abilities of all the drugs used; moreover, this coumarin enhances neuroprotective effects of PB as well as VPA in an excitotoxicity model of neurodegeneration. 

UMB’s synergistic effect on investigational drugs may be important in the context of epilepsy patient safety, as it is known that PB and VPA cause apoptotic neurodegeneration in the developing rat brain [3]. Neuronal death is associated with reduced expression of neurotrophins and decreased concentrations of survival-promoting proteins in the brain. ASMs depress the endogenous neuroprotective system in the brain that is crucial for neuronal survival during development [3,75]. PB and VPA depressed the synthesis of the neurotrophins BDNF and NT-3 and reduced the levels of the active phosphorylated forms of c-RAF, ERK1/2, and AKT. Such changes reflect the impairment of survival-promoting signals and an imbalance between neuroprotective and neurodestructive mechanisms in the brain; during the developmental period of ongoing programmed neuronal death, this will promote apoptotic neurodegeneration [76].

## 4. Materials and Methods

### 4.1. In Vivo Study

#### 4.1.1. Animals and Experimental Conditions

The experiments were conducted on adult male albino Swiss mice (weighing 22–26 g) that were kept in colony cages with free access to food and tap water, under standardized housing conditions. After 7 days of adaptation, the animals were randomly assigned to experimental groups consisting of 8 mice. The experimental procedures described herein were approved by the Local Ethics Committee for Animal Experimentation at the University of Life Sciences in Lublin, Poland (License no.: 54/2020 and 73/2021). They comply with the ARRIVE guidelines and were conducted in strict accordance with the EU Directive 2010/63/EU for animal experiments. All efforts were made to minimize animal suffering and to use only the number of animals necessary to produce reliable scientific data according to the 3Rs rule [77].

#### 4.1.2. Drugs

Umbelliferone (UMB, Sigma-Aldrich, Poznan, Poland), lacosamide (LCM, UCB Pharma, Brussels, Belgium), levetiracetam (LEV, UCB Pharma, Braine-l’Alleud, Belgium), phenobarbital (PB, Polfa, Krakow, Poland), and valproate (VPA, sodium salt—Sigma-Aldrich, Poznan, Poland) were used in this study. All substances, except for valproate, were suspended in a 1% solution of Tween 80 (Sigma, St. Louis, MO, USA) in distilled water, while VPA was directly dissolved in distilled water only. All anti-seizure medications (ASMs) were administered intraperitoneally (i.p.) via injection, in a volume of 5 mL/kg body wt, as follows: LCM and VPA—30 min; LEV and PB—60 min, before initiation of 6-Hz corneal-stimulation-induced seizures, motor coordination, grip-strength and long-term memory tests, as well as before brain sampling for the measurement of antiepileptic drug concentrations. The UMB was administered i.p. 30 min before all experiments. The time-points of UMB and ASMs were based upon information about their biological activity from the literature, and on our previous experiments [12,15]. UMB doses were established based on previous studies, where this coumarin administered at a dose of 100 mg/kg did not significantly affect the threshold for electroconvulsions in mice in the maximal electroshock seizure threshold test [12]. Moreover, for ethical reasons, in accordance with the 3Rs rule, the 6 Hz-induced seizure threshold test (which would require at least an additional 128 mice) was not performed.

#### 4.1.3. 6-Hz Corneal-Stimulation-Induced Seizures

Seizure activity in mice was evoked by a current (6 Hz, 0.2 ms rectangular pulse width, 32 mA, 3 s duration) generated by an S48 Square Pulse Stimulator and CCU1 Constant Current Unit (Grass Technologies, West Warwick, RI, USA). After the application of an ocular anesthetic (0.5% solution of tetracaine hydrochloride) to the mouse corneas, the animals underwent corneal stimulation and were placed separately in Plexiglas cages (25 cm × 15 cm ×10 cm) for the observation of the presence or absence of psychomotor seizures, as described previously [15,78,79]. When the observation of 8 mice in the respective group was finished, the animals underwent euthanasia by CO_2_ narcosis. To determine median effective doses (ED_50_ values) of ASMs, the drugs were administered i.p. at the following doses: LCM: 2–10 mg/kg; LEV: 10–20 mg/kg; PB: 2–12 mg/kg and VPA: 50–150 mg/kg.

#### 4.1.4. Chimney Test

The influence of ASMs administered alone, and in combination (in doses reflecting their ED_50_ values from the 6-Hz corneal stimulation model) with UMB (100 mg/kg, administered 30 min before the test) on motor coordination in mice was determined using the chimney test, as described elsewhere [15,80].

#### 4.1.5. Grip-Strength Test

The influence of ASMs administered alone, and in combination (in doses reflecting their ED_50_ values from the 6-Hz corneal stimulation model) with UMB (100 mg/kg, administered 30 min before the test) on the muscular strength of the forelegs in mice was determined using the grip-strength test, as described elsewhere [15,80].

#### 4.1.6. Passive Avoidance Task

The influence of ASMs administered alone, and in combination (in doses reflecting their ED_50_ values from the 6-Hz corneal stimulation model) with UMB (100 mg/kg, administered 30 min before the test), on long-term memory (acquisition, learning and remembering) in mice was determined using a passive avoidance task, as described in detail elsewhere [15,80].

#### 4.1.7. Measurement of Total Brain Antiepileptic Drug Concentrations

Measurements of total brain concentrations of PB (at a dose of 3.3 mg/kg) and VPA (at a dose of 74.6 mg/kg) were undertaken in the mice receiving either the antiepileptic drugs alone or the combination of ASM with UMB (at a dose of 100 mg/kg). Mice were killed by decapitation at times corresponding to the peak of maximum anticonvulsant effects for the antiepileptic drugs in the 6 Hz test. The whole brains of mice were removed from the skulls, weighed, and homogenized using an Abbott buffer (1:2 *w*/*v*) in an Ultra-Turrax T8 homogenizer (IKA Werke, Staufen, Germany). The homogenates were then centrifuged at 10,000× *g* for 10 min, and the supernatant samples of 200 μL were collected. 

The PB and VPA concentrations from the brains were analyzed with high-performance liquid chromatography (HPLC). The HPLC system consisted of a UV/VIS detector (UVD 340S), a gradient pump P580 LPG, and a Rheodyne 3601 injector valve with a 20 µL sample loop (Dionex, Sunnyvale, CA, USA). Brain PB concentrations were analyzed with A HyPURITY C18 column (150 mm × 4.6 mm, 5 μm particle size) using a mobile phase of 50 mM phosphate buffer solution containing 20% acetonitrile at pH 3.8.A modified method by Lukawski et al. (2018) was employed for determination of VPA [81]. The chromatographic separation of VPA was achieved using a reversed phase ODS-2 Hypersil column (150 mm × 4.6 mm, 5 μm particle size) with a mobil phase consisting of 40 mM phosphate buffer solution (pH 3.0) acetonitrile-isopropanol (60:25:15 *v*/*v*/*v*). Chromatography was performed at ambient temperature using a flow ratio of 0.8 mL/min. The column eluates were monitored at 225 nm for PB and at 360 nm for VPA, with a sensitivity of 0.01 absorbance units full scale (AUFS), and a time constant of 0.1 s.

Total brain concentrations of PB and VPA were expressed in μg/g of wet brain tissue as means ± standard error (SEM) of at least 6 separate brain preparations.

### 4.2. In Vitro Study

#### 4.2.1. Reagents

All reagents and kits were purchased from Sigma-Aldrich (St. Louis, MO, USA), unless otherwise indicated.

#### 4.2.2. Cell Line

The human undifferentiated neuroblastoma cell line SH-SY5Y was purchased from ECACC (European Collection of Cell Cultures, Salisbury, UK.). Cells were grown in a 1:1 mixture of Ham’s F12 nutrient and Dulbecco’s Modified Eagle Medium (DMEM/F12) supplemented with 10% fetal bovine serum (FBS), 1% non-essential amino acid solution, penicillin (100 U/mL) and streptomycin (100 mg/mL). Cells were maintained in a humidified atmosphere of 95% air and 5% CO_2_ at 37°C (standard conditions). In order to differentiate neuroblastoma cells from neurons, SH-SY5Y cells at a density of 1 × 106 cells/mL were resuspended in Alpha MEM Gluta MAX medium supplemented with 0.5 μM retinoic acid. Afterwards, cells were seeded on a low-attachment Petri dish and incubated for 5 days in standard conditions. The resulting neurospheres were collected and treated with 0.05% trypsin-EDTA for 15 min in 37°C. After blocking trypsin with FBS, cells were shaken in the presence of 100 μg/mL DNAase I. Finally, the cell suspension was centrifuged at 1200 rpm for 5 min and resuspended in Neurobasal medium (Gibco) containing 2% B-27 supplement (Gibco), 2 mM L-glutamine, penicillin (100 U/mL), and streptomycin (100 mg/mL). The cells were seeded on 96-well microplates (Nunc) coated with poly-D-lysine at a density of 1 × 105 cells/mL and kept under standard conditions. The culture medium was changed every 4 days. All experiments were carried out after 12 days of culture.

#### 4.2.3. Cell Viability Assessment—MTT Assay

Neurons cultured for 12 days were exposed to serial dilutions of UMB (1, 2.5, 5 µg/mL), LCM, LEV, PB, and VPA (1, 2.5, 5 µg/mL), and combined with UMB (1 µg/mL). In the excitotoxicity model of neurodegeneration, 5 mM glutamate was added to the culture. Solutions of investigated compounds were prepared in the Neurobasal medium supplemented with 2% B-27, 2 mM L-glutamine, penicillin (100 U/mL) and streptomycin (100 mg/mL). In the case of experiments performed in conditions of trophic stress, solutions of all drugs were prepared in the medium deprived of a B-27 supplement. In the combinations of tested drugs with UMB, a dose of 1 µg/mL of this coumarin was used because it did not significantly increase the viability of nerve cells in both models of neurodegeneration (Figure 3).

After 48 h of cells exposure to the investigated compounds, MTT solution (5 mg/mL in PBS) was added for 6 h. The resultant crystals were solubilized overnight in SDS buffer with a pH of 7.4 (10% SDS in 0.01 N HCl) and the product quantified spectrophotometrically by measuring the absorbance at a 570 nm wavelength using a microplate reader (BioTek ELx800, Highland Park, Winooski, VT, USA). The results were presented as a percentage of cell viability treated with the investigated compounds versus cells grown in the control medium (indicated as 100%).

### 4.3. Statistical Analyses

The ED_50_ values for antiepileptic drugs determined in the 6-Hz corneal stimulation model were calculated by computer log-probit analysis [82], as described in detail earlier [83]. Subsequently, the ED_50_ values (±SEM) were statistically analyzed using one-way analysis of variance (ANOVA) followed by the post-hoc Tukey–Kramer test for multiple comparisons. Total brain ASM concentrations and the results of the grip-strength test were statistically verified using one-way analysis of variance (ANOVA). The results of the chimney test were compared using Fisher’s exact probability test. The results of the passive avoidance task were statistically analyzed using the nonparametric Kruskal–Wallis test. The data from the in vivo study were presented as the mean value and standard error of the mean (SEM). Statistical analysis was performed using one-way ANOVA with the Tukey post-hoc test, and column statistics were used for comparisons. The differences among values were considered statistically significant if *p* < 0.05. GraphPad Prism version 8.0 for Windows (GraphPad Software, San Diego, CA, USA) was used as the statistical software.

## 5. Conclusions

The conducted research revealed that the use of UMB in combination with PB and VPA is beneficial from a clinical point of view, as it lacks side effects and adverse pharmacokinetic interactions. In addition, UMB enhanced the neurotrophic abilities of all the drugs used in the study. Furthermore, this coumarin enhances the neuroprotective effects of PB as well as VPA in an excitotoxicity model of neurodegeneration. However, further research is required to thoroughly understand the mechanism of UMB’s molecular activity and to assess the long-term safety of its clinical use.

## Figures and Tables

**Table 1:** Effects of UMB and its combinations with varipus anti-seizure medications on long-term memory, muscular strength and motor performance in mice.

Drug [mg/kg]	Retention Time (s)	Grip Strength (gf)	Motor Coordination Impairment (%)
Vehicle	180 (115; 180)	125.8 ± 4.99	0
LCM (3.6)	180 (71; 180)	120.0 ± 1.25	0
LCM (3.6) + UMB (100)	142 (81; 180)	112.6 ± 3.86	25
LEV (11.9)	180 (66; 180)	114.7 ± 4.55	0
LEV (11.9) + UMB (100)	180 (55; 180)	108.4 ± 7.43	25
PB (3.3)	180 (59; 180)	122.9 ± 1.87	0
PB (3.3) + UMB (100)	129 (68; 180)	121.1 ± 3.69	0
VPA (74.6)	171 (49; 180)	118.0 ± 2.58	0
VPA (74.6) + UMB (100)	180 (51; 180)	115.1 ± 5.69	0

Results are presented as: (1) median retention times (in seconds, with 25th and 75th percentiles in parentheses) from the passive avoidance task, assessing long-term memory in mice; (2) mean grip-strengths (in gfs ± SEM) from the grip-strength test, assessing muscular strength in mice; and (3) percentage of animals showing motor coordination impairment in the chimney test in mice. Each experimental group consisted of 8 mice. Statistical analysis of data from the passive avoidance task was performed with nonparametric Kruskal–Wallis ANOVA test, whereas those from the grip-strength test were analyzed with one-way ANOVA followed by Bonferroni’s post-hoc test. Fisher’s exact probability test was used to analyze the results from the chimney test. All drugs were administered intraperitoneally (i.p.) at times scheduled from the 6-Hz corneal-stimulation-induced seizures and at doses corresponding to their ED_50_ values against 6-Hz test.

## Data Availability

The data supporting reported results can be found in the laboratory databases of Institute of Rural Health.
